# Supplementary material for: A Novel RNA Virus in the Parasitoid Wasp Lysiphlebus fabarum: Genomic Structure, Prevalence, and Transmission
Source: Viruses. 2020 Jan 3;12(1):59. doi: 10.3390/v12010059 (PMC7019493; doi:10.3390/v12010059)
Supplement: Supplementary file 1 [file viruses-12-00059-s001.pdf]

Figure S1: Transmission assay results: Only H76 wasps are shown for the three pools with viral detection in generation 3 (all female-female pairs). qPCR allows viral detection in all three generations of all shown pools, however vertical transmission was not perfect as not all offspring in generation 3 carry the virus (pool 6). Bars represent viral RNA normalized to GAPDH ± standard error. These values are shown on a log scale. Generations are coded by color: generation 1 (blue), generation 2 (turquoise) and generation 3 (light blue). Asterisks show which samples were negative for PCR but positive for qPCR and the dashed line depicts where viral load equals that of GAPDH.

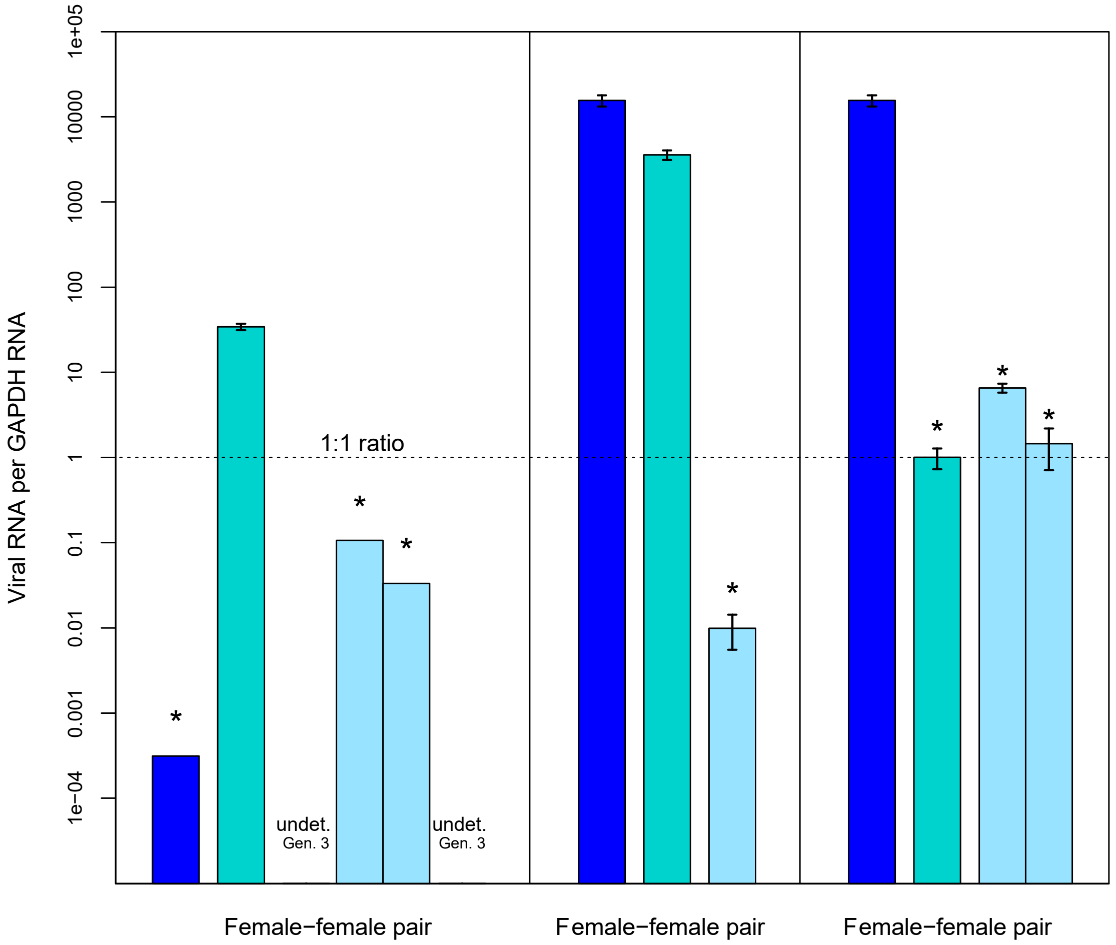

Figure S2: Amino acid variation between the two whole-genome sequences of LysV types A and B. Predicted proteins from the whole-genome assembly are noted with black boxes: 5`UTR, RdRp, and two of the structural capsid proteins (the first and third), marked with #.

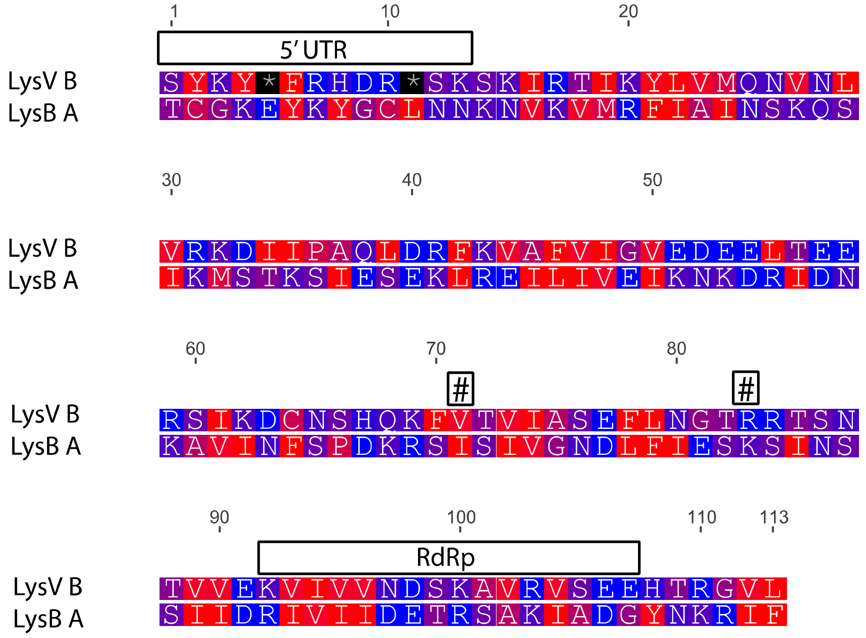

Figure S3: Efficiencies are shown for cDNA samples at two primer concentrations (1 and 5 µM) for the virus and GAPDH target, determined by the slope of the linear regression models. All efficiency values are close to the optimal value and the values for the targets of the same primer concentration do not differ by more than 10%. The primer concentration of 5 µM was chosen for all qPCR screenings.

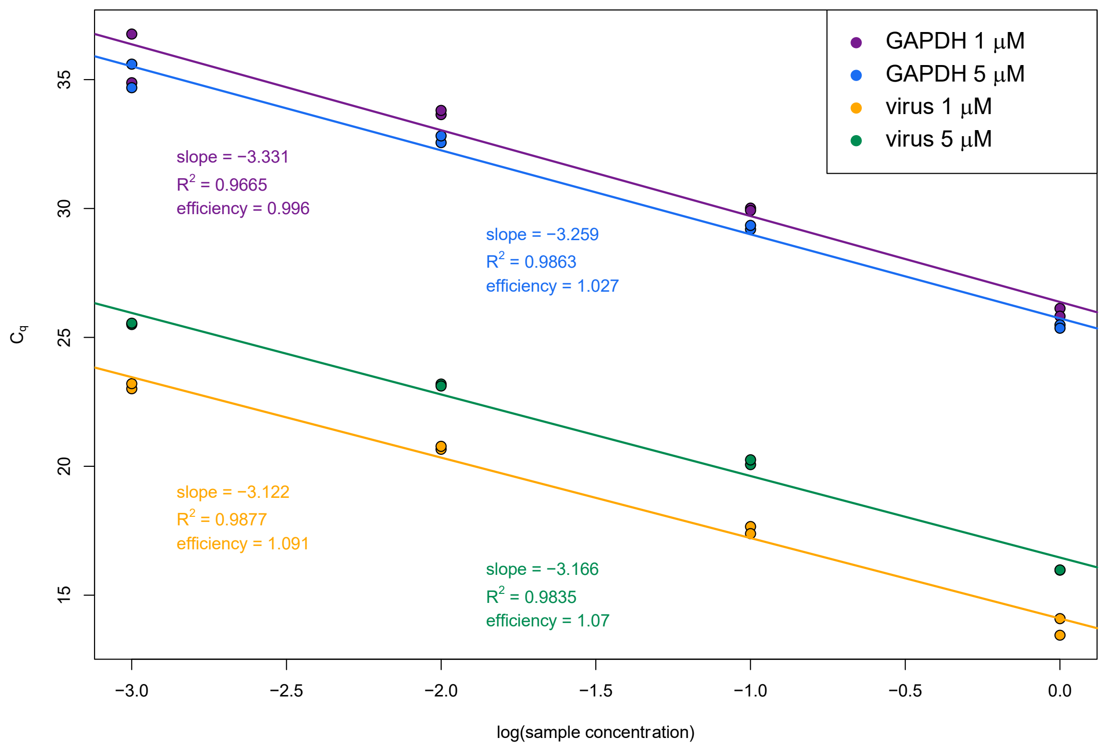

Table S1: Aphids and wasps transferred to *V. faba* plants for rearing of experimental lines.

L. fabarum line	*A. fabae* line	*A. fabae* genotype	Treatment name	Number of replicate plants
H-	H-	A06-407	H-	3
H76	H76	A06-407	H76	3
H76	H-	A06-407	H76 on H-	6
H402	H402	A06-407	H402	3
H402	H-	A06-407	H402 on H-	6

Table S2*:* Wild-caught parasitoids sampled throughout Europe and screened for LysV. Asterisks indicate viral presence in a sample.

Yea	Sample ID	Area	Plant species	Aphid species	*Lysiphlebus fabarum* morphotype[1]	Sex	# of wasps tested	Founding population experimental evolution?	Population sample type
200	06–11	Sarzana, Toscana	*Vicia faba*	*Aphis fabae fabae*	*L. fabarum*	F/M	5	no	wild
200	06–13	Sarzana, Toscana	*Vicia faba*	*Aphis fabae fabae*	*L. fabarum*	F	5	no	wild
200	06–16	Carrara, Toscana	*Rubus fruticosus*	*Aphis ruborum*	*L. fabarum*	F/M	5	no	wild
200	06–30	Cesena, Romagna	*Vicia faba*	*Aphis fabae fabae*	*L. confusus*	F	5	no	wild
200	06–45	Lunel, Camargue	*Rubus fruticosus*	*Aphis ruborum*	*L. confusus*	F	5	no	wild
200	06–94	Port Grimaud, Côte d'Azur	*Rumex sp.*	*Aphis rumicis*	*L. confusus*	F	5	no	wild
200	06–115	Le Muy, Côte d'Azur	*Lactuca sp.*	*Aphis sp.*	*L. fabarum*	F/M	5	no	wild
200	06–116	Le Muy, Côte d'Azur	*Carduus tenuiflorus*	*Brachycaudus cardui and Aphis fabae cirsiicanthoides*	*L. confusus*	F	5	no	wild
200	06–118	Le Muy, Côte d'Azur	*Vicia faba*	*Aphis fabae fabae*	*L. confusus*	F	5	no	wild
200	06–120	Draguignon, Côte d'Azur	*Hedera helix*	*Aphis hederae*	*L. fabarum*	M	5	no	wild
200	06–153	Martigny, Valais	*Cirsium arvense*	*Aphis fabae cirsiiacanthoides*	*L. confusus*	F	5	no	wild
200	06–159	Martigny, Valais	*Cirsium arvense*	*Aphis fabae cirsiiacanthoides*	*L. fabarum*	F/M	5	no	wild
200	06–175	Pont de la Marge, Valais	*Hedera helix*	*Aphis hederae*	*L. fabarum*	F/M	5	no	wild
200	06–181	Pont de la Merge, Valais	*Hedera helix*	*Aphis hederae*	*L. fabarum*	F	5	no	wild
200	06–197	Sion, Valais	*Rubus fruticosus*	*Aphis ruborum*	*L. confusus*	F	5	no	wild
200	06–201	Sion, Valais	*Cirsium arvense*	*Aphis fabae cirsiiacanthoides*	*L. fabarum*	F	5	no	wild
200	06–202	Sion, Valais	*Urtica dioica*	*Aphis urticata*	*L. confusus*	F	5	no	wild
200	06–204	Sion, Valais	*Rubus fruticosus*	*Aphis ruborum*	*L. confusus*	F	5	no	wild
200	06–216	S. Antonio, Ticino	*Rubus fruticosus*	*Aphis ruborum*	*L. confusus*	F	5	no	wild
200	06–236	Quartino, Ticino	*Rumex sp.*	*Aphis rumicis*	*L. cardui*	F	5	no	wild
200	06–240	Quartino, Ticino	*Rubus fruticosus*	*Aphis ruborum*	*L. cardui*	F	5	no	wild
200	06–244	Quartino, Ticino	*Chenopodium album*	*Aphis fabae fabae*	*L. fabarum*	F	5	no	wild
200	06–245	Quartino, Ticino	*Urtica dioica*	*Aphis urticata*	*L. fabarum*	F/M	5	no	wild
200	06–277	Vezin, Bretagne	*Rubus fruticosus*	*Aphis ruborum*	*L. fabarum*	F	5	no	wild

200	06–307	Chur, Chur	*Rubus fruticosus*	*Aphis ruborum*	*L. confusus*	F	5	no	wild
200	06–343	Budweis, Czech Republic	*Chenopodium album*	*Aphis fabae fabae*	*L. fabarum*	F/M	5	no	wild
200	06–364	Budweis, Czech Republic	*Rumex sp.*	*Aphis rumicis*	*L. cardui*	F	5	no	wild
200	06–375	Lomnice, Czech Republic	*Cirsium arvense*	*Aphis fabae cirsiiacanthoides*	*L. fabarum*	F/M	5	no	wild
200	06–377	Vezeli, Czech Republic	*Cirsium arvense*	*Aphis fabae cirsiiacanthoides*	*L. cardui*	F	5	no	wild
200	06–389	Novosedly, Czech Republic	*Salix sp.*	*Aphis farinosa*	*L. confusus*	F	5	no	wild
200	06–394	Budweis, Czech Republic	*Salix sp.*	*Aphis farinosa*	*L. cardui*	F	5	no	wild
200	06–412	Straz, Czech Republic	*Cirsium arvense*	*Aphis fabae cirsiiacanthoides*	*L. cardui*	F	5	no	wild
200	06–426	Steinmaur, Zürich	*Rubus fruticosus*	*Aphis ruborum*	*L. confusus*	F	5	no	wild
200	06–438	Aesch Birspark, Basel	*Hedera helix*	*Aphis hederae*	*L. fabarum*	F/M	5	no	wild
200	06–451	Aesch, Basel	*Vicia faba*	*Aphis fabae fabae*	*L. cardui*	F	5	no	wild
200	06–466	Arlesheim, Basel	*Hedera helix*	*Aphis hederae*	*L. fabarum*	F	5	no	wild
200	06–480	Liestal, Basel	*Cirsium arvense*	*Aphis fabae cirsiicanthoides*	*L. cardui*	F	5	no	wild
200	06–486	Liestal, Basel	*Hedera helix*	*Aphis hederae*	*L. fabarum*	F	5	no	wild
200	06–500	Hailer Industrie, Hessen	undetermined	*Aphis fabae cirsiiacanthoides*	*L. fabarum*	F/M	5	no	wild
200	06–509	Altenhasslau Industrie, Hessen	*Chenopodium album*	*Aphis fabae fabae*	*L. fabarum*	F	5	no	wild
200	06–544	Elmshorn, North Sea	*Cirsium arvense*	*Aphis fabae cirsiicanthoides*	*L. cardui*	F	5	no	wild
200	06–549	Elmshorn, North Sea	*Rubus fruticosus*	*Aphis ruborum*	*L. fabarum*	F	5	no	wild
200	06–556	Elmshorn, North Sea	*Rubus fruticosus*	*Aphis ruborum*	*L. fabarum*	F/M	5	no	wild
200	06–585	Elmshorn, North Sea	*Cirsium arvense*	*Aphis fabae crisiiacanthoides*	*L. cardui*	F	5	no	wild
200	06–659	Madingley, Cambridge	*Vicia faba*	*Aphis fabae fabae*	*L. fabarum*	F/M	5	no	wild
200	06–674	Bar Hill, Cambridge	*Rubus fruticosus*	*Aphis ruborum*	*L. confusus*	F	5	no	wild
200	06–705	Glückstadt, North Sea	*Hedera helix*	*Aphis hederae*	*L. fabarum*	F	5	no	wild
200	06–744	Friedrichskoog Spitze, North Sea	*Cirsium arvense*	*Aphis fabae cirsiiacanthoides*	*L. cardui*	F	5	no	wild
200	06–747	Meldorf, North Sea	*Hedera helix*	*Aphis hederae*	*L. fabarum*	F	5	no	wild
200	06–756	Heide, North Sea	*Cirisum arvense*	*Aphis fabae cirsiiacanthoides*	*L. cardui*	F	5	no	wild

200	09–89	Romans, Valence	*Hedera helix*	*Aphis hederae*	*L. fabarum*	F	5	no	wild
200	09–108	Romans, Valence	*Hedera helix*	*Aphis hederae*	*L. fabarum*	M	5	no	wild
200	09–222	Martigny, Valais	*Hedera helix*	*Aphis hederae*	*L. fabarum*	F	5	no	wild
200	09–223	Martigny, Valais	*Hedera helix*	*Aphis hederae*	*L. fabarum*	M	5	no	wild
200	09–226	Martigny, Valais	*Hedera helix*	*Aphis hederae*	*L. fabarum*	F	5	no	wild
200	09–227	Martigny, Valais	*Hedera helix*	*Aphis hederae*	*L. fabarum*	F	5	no	wild
200	09–372	Orbe, Vaud	*Hedera helix*	*Aphis hederae*	*L. fabarum*	M	5	no	wild
200	09–377	Orbe, Vaud *	*Hedera helix*	*Aphis hederae*	*L. fabarum*	F	5	no	wild
201	12–9	Regensberg, Zürich	*Hedera helix*	*Aphis hederae*	*L. fabarum*	F/M	3	no	before maintenance in lab
201	12–10	Regensberg, Zürich	*Hedera helix*	*Aphis hederae*	*L. fabarum*	F/M	3	no	before maintenance in lab
201	12–11	Regensberg, Zürich	*Hedera helix*	*Aphis hederae*	*L. fabarum*	F/M	3	**founding population**	before maintenance in lab
201	12–62	Fribourg, Fribourg *	*Hedera helix*	*Aphis hederae*	*L. fabarum*	F/M	1	**founding population**	before maintenance in lab
201	12–80	Geneva, Geneva	*Hedera helix*	*Aphis hederae*	*L. fabarum*	F/M	1	no	before maintenance in lab
201	12–81	Geneva, Geneva	*Hedera helix*	*Aphis hederae*	*L. fabarum*	F/M	3	**founding population**	before maintenance in lab
201	12–83	Geneva, Geneva	*Hedera helix*	*Aphis hederae*	*L. fabarum*	F/M	3	**founding population**	before maintenance in lab
201	12–84	Geneva, Geneva	*Hedera helix*	*Aphis hederae*	*L. fabarum*	F/M	1	no	before maintenance in lab
201	12–87	Nyon, Vaud	*Hedera helix*	*Aphis hederae*	*L. fabarum*	F/M	3	**founding population**	before maintenance in lab
201	12–89	Nyon, Vaud *	*Hedera helix*	*Aphis hederae*	*L. fabarum*	F/M	3	**founding population**	before maintenance in lab
201	12–93	Nyon, Vaud	*Hedera helix*	*Aphis hederae*	*L. fabarum*	F/M	3	no	before maintenance in lab
201	12–101	Renens, Vaud	*Hedera helix*	*Aphis hederae*	*L. fabarum*	F/M	1	**founding population**	before maintenance in lab
201	12–102	Renens, Vaud	*Hedera helix*	*Aphis hederae*	*L. fabarum*	F/M	1	no	before maintenance in lab
201	12–11	Regensberg, Zürich	*Hedera helix*	*Aphis hederae*	*L. fabarum*	F/M	4	**founding population**	after maintenance in lab
201	12–51	Maladière, Lausanne	*Hedera helix*	*Aphis hederae*	*L. fabarum*	F/M	4 (combined with 12–56)	**founding population**	after maintenance in lab
201	12–55	Maladière, Lausanne	*Hedera helix*	*Aphis hederae*	*L. fabarum*	F/M	4	**founding population**	after maintenance in lab

201	12–56	Maladière, Lausanne	*Hedera helix*	*Aphis hederae*	*L. fabarum*	F/M	4 (combined with 12–51)	**founding population**	after maintenance in lab
201	12–62	Fribourg, Fribourg	*Hedera helix*	*Aphis hederae*	*L. fabarum*	F/M	4 (combined with 12–83)	**founding population**	after maintenance in lab
201	12–69	Fribourg, Fribourg	*Hedera helix*	*Aphis hederae*	*L. fabarum*	F/M	4	**founding population**	after maintenance in lab
201	12–81	Geneva, Geneva	*Hedera helix*	*Aphis hederae*	*L. fabarum*	F/M	4	**founding population**	after maintenance in lab
201	12–83	Geneva, Geneva	*Hedera helix*	*Aphis hederae*	*L. fabarum*	F/M	4 (combined with 12–62)	**founding population**	after maintenance in lab
201	12–87	Nyon, Vaud	*Hedera helix*	*Aphis hederae*	*L. fabarum*	F/M	4 (combined with 12–92)	**founding population**	after maintenance in lab
201	12–89	Nyon, Vaud *	*Hedera helix*	*Aphis hederae*	*L. fabarum*	F/M	4	**founding population**	after maintenance in lab
201	12–92	Nyon, Vaud	*Hedera helix*	*Aphis hederae*	*L. fabarum*	F/M	4 (combined with 12–87)	**founding population**	after maintenance in lab
201	12–101	Renens, Vaud *	*Hedera helix*	*Aphis hederae*	*L. fabarum*	F/M	4	**founding population**	after maintenance in lab
201	16–9	Sünikon, Zürich	*Hedera helix*	*Aphis hederae*	*L. fabarum*	F	1	no	wild
201	16–12	Zürich, Zürich	*Hedera helix*	*Aphis hederae*	*L. fabarum*	F	1	no	wild
201	16–16	Dübendorf, Zürich	*Rubus fruticosus*	*Aphis ruborum*	*L. fabarum*	F	1	no	wild
201	16–24	Dübendorf, Zürich	*Salix caprea*	*Aphis farinosa*	*L. confusus*	F	1	no	wild
201	16–25	Dübendorf, Zürich	*Hedera helix*	*Aphis hederae*	*L. fabarum*	F	1	no	wild
201	16–32	Zug, Zug	*Hedera helix*	*Aphis hederae*	*L. fabarum*	F	1	no	wild
201	16–33	Wallisellen, Zürich	*Cirsium vulgare*	*Brachycaudus cardui*	*L. fabarum*	F	1	no	wild
201	16–34	Wallisellen, Zürich	*Rubus fruticosus*	*Aphis ruborum*	*L. confusus*	F	1	no	wild
201	16–48	Dübendorf, Zürich	*Salix sp.*	*Aphis farinosa*	*L. confusus*	F	1	no	wild
201	16–49	Dübendorf, Zürich	*Urtica dioica*	*Aphis urticata*	*L. cardui*	F	1	no	wild
201	16–52	Dübendorf, Zürich	*Rubus fruticosus*	*Aphis ruborum*	*L. confusus*	F	1	no	wild
201	16–55	Dielsdorf, Zürich	*Salix caprea*	*Aphis farinosa*	*L. confusus*	F	1	no	wild
201	16–66	Dübendorf, Zürich	*Cirsium vulgare*	*Brachycaudus cardui*	*L. fabarum*	F	1	no	wild

[1] Parasitoids of the *Lysiphlebus fabarum* group exhibit some morphological variation that has traditionally been subsumed under three distinct taxonomic names (*L. fabarum*, *L. confusus*, and *L. cardui*), but more recent evidence indicates that they should be treated as conspecific morphotypes [1].

Table S3*:* Viral taxa included in the phylogenetic analysis based on the RdRP gene. All families shown belong to the *Picornavirales* order. Formatted sequence data were kindly provided by Dr. Dheilly [2].

Abbreviation	Virus species	Genbank Number	Family
ABPV	Acute bee paralysis virus	NC_002548	*Dicistroviridae*
ALPV	Aphid lethal paralysis virus	NC_004365	*Dicistroviridae*
BQCV	Black queen cell virus	NC_003784	*Dicistroviridae*
DCV	*Drosophila* C virus	NC_001834	*Dicistroviridae*
HCV	*Homalodisca coagulata* virus–1	NC_008029	*Dicistroviridae*
HPV	*Himetobi* P virus	NC_003782	*Dicistroviridae*
MCD	Mud crab dicistrovirus	NC_014793	*Dicistroviridae*
PSIV	*Plautia stali* intestine virus	NC_003779	*Dicistroviridae*
RPV	*Rhopalosiphum padi* virus	NC_001874	*Dicistroviridae*
SIV	*Solenopsis invicta* virus	NC_006559	*Dicistroviridae*
TSV	Taura syndrome virus	JX094350	*Dicistroviridae*
TV	*Triatoma* virus	NC_003783	*Dicistroviridae*
BbPLV	*Brevicoryne brassicae* picorna–like virus	NC_009530	*Iflaviridae*
BSPV	Bee slow paralysis virus	EU_035616	*Iflaviridae*
DcPV	*Dinocampus coccinellae* paralysis virus	KF843822	*Iflaviridae*
DWV	Deformed wing virus	NC_004830	*Iflaviridae*
EoV	*Extropis obliqua* virus	NC_005092	*Iflaviridae*
IFV	Infectious flatcherie virus	AB_000906	*Iflaviridae*
KV	Kakugo virus	NC_005876	*Iflaviridae*
LlV–1	*Lygus lineolaris* virus–1	JF_720348	*Iflaviridae*
NlHV–1	*Nilaparvata lugens* honeydew virus	AB_766259	*Iflaviridae*
NvitV–1	*Nasonia vitripennis* virus isolate 1	FJ790486	*Iflaviridae*
PnV	*Perina nuda* virus	AF323747	*Iflaviridae*
SBV	Sacbrood virus	NC_002066	*Iflaviridae*
SeIV–1	*Spodoptera exigua* Iflavirus–1	JN_091707	*Iflaviridae*
SeIV–2	*Spodoptera exigua* Iflavirus–2	JN_870848	*Iflaviridae*
VcPLV	*Venturia canescens* picorna–like virus	AY534885	*Iflaviridae*
VDV	*Varroa destructor* virus	NC_006494	*Iflaviridae*
EMCV	Encephalomyocarditis virus	NC_001479	*Picornaviridae*
Poliovirus	Poliovirus	NC_002058	*Picornaviridae*

Table S4: Fisher's exact test p-values (rejection of the null hypothesis for all tested lines) showing that frequencies of infection were unequal among parasitoid lines.

Lines tested	# of lines in grouping	p–value	Independency hypothesis rejected?
Lab populations	10	< 0.001	reject
Evolution lines	5	0.016	reject
Infected lab populations	3	< 0.001	reject
Transmission assay	9	< 0.001	reject
Wild populations	6	< 0.001	reject

Table S5: Mixed models testing for differences of viral infection intensity as estimated from qPCR, comparing either individuals of lab populations (top half) or ovary dissected samples of lab populations (bottom half). Only lines with samples present in both April and October 2016 were evaluated.

Source	ndf for fixed effects	ddf for fixed effects	F for fixed effects / LR χ^2_1 for random effects	p-value
**Wasp individuals (April and October 2016)**				
**Sexual population**				
Time point	1	5	0.071	0.801
Sample in time point			66.507	< 0.001
**H76**				
Time point	1	1	32.474	0.111
Sample in time point			7.107	0.008
**H402**				
Time point	1	2	4.827	0.159
Sample in time point	1 sample per time point, fixed effects model only			
**Ovary dissections (April and October 2016)**				
**H76**				
Time point	1	1	0.404	0.640
Sample in time point			19.894	< 0.001
**H76 on H-**				
Time point	1	1	1.634	0.422
Sample in time point			16.453	< 0.001
**H402 on H-**				
Time point	1	2	4.079	0.174
Sample in time point			5.843	0.016

Table S6: Mixed model comparing either generation 3 individuals of pools with detected horizontal transmission, based on qPCR values (top), infected H76 individuals of all three generations for vertical transmission (middle) or H76 individuals with vertical transmission across two generations (bottom).

Source	ndf for fixed effects	ddf for fixed effects	F for fixed effects / LR χ^2_1 for random effects	p-value
**Horizontal transmission generation 3 individuals**				
**Pool 4 (female-female pair)**				
Wasp line	1	3	4.337	0.129
Sample in wasp line			35.601	< 0.001
**Pool 14 (female-female pair)**				
Wasp line	1	12	9.863	0.009
Sample in wasp line			131.690	< 0.001
**Pool 20 (large pool)**				
Wasp line	1	1	0.098	0.807
Sample in wasp line			33.180	< 0.001
**H76 individuals in pools with vertical transmission across 3 generations**				
**Pool 6 (female-female pair)**				
Generation	1	2	3.857	0.188
Sample in generation			11.043	< 0.001
**Pool 19 (female-female pair)**				
Generation	1	1	0.500	0.608
Sample in generation			27.412	< 0.001
**Pool 31 (female-female pair)**				
Generation	1	2	52.834	0.018
Sample in generation			6.802	0.009
**H76 individuals in pools with vertical transmission across 2 generations**				
**H76 replicate 1**				
Generation	1	4	19.0	0.012
Sample in generation	1 sample per generation, fixed effects model only			
**H76 replicate 7**				
Generation	1	4	24.694	0.008
Sample in generation			51.077	< 0.001
**H76 replicate 8**				
Generation	1	6	0.212	0.662
Sample in generation			50.190	< 0.001

References:

1. Tomanović, Ž.; Mitrović, M.; Petrović, A.; Kavallieratos, N. G.; Žikić, V.; Ivanović, A.; Rakhshani, E.; Starý, P.; Vorburger, C., Revision of the European Lysiphlebus species (Hymenoptera: Braconidae: Aphidiinae) on the basis of COI and 28SD2 molecular markers and morphology. *Arthropod Syst Phylo* **2018,** 76, 179-213.
2. Dheilly, N. M.; Poulin, R.; Thomas, F., Biological warfare: Microorganisms as drivers of host-parasite interactions. *Infect Genet Evol* **2015,** 34, 251-9.
